# Efficacy and Safety of Tenofovir Disoproxil Fumarate Versus Low-Dose Stavudine Over 96 Weeks: A Multicountry Randomized, Noninferiority Trial

**DOI:** 10.1097/QAI.0000000000001908

**Published:** 2018-11-12

**Authors:** Willem Daniel Francois Venter, Andrew Kambugu, Matthew F. Chersich, Stephen Becker, Andrew Hill, Natasha Arulappan, Michelle Moorhouse, Mohammed Majam, Godspower Akpomiemie, Simiso Sokhela, Selvamuthu Poongulali, Charles Feldman, Chris Duncombe, David H. Brown Ripin, Alinda Vos, Nagalingeswaran Kumarasamy

**Affiliations:** * Wits Reproductive Health and HIV Institute, Faculty of Health Sciences, University of the Witwatersrand, Johannesburg, South Africa;; † Infectious Diseases, College of Health Sciences at Makerere University, Kampala, Uganda;; ‡ Independent Consultant, Yountville, CA;; § Pharmacology Department, University of Liverpool, Liverpool, United Kingdom;; ║ Chennai Antiviral Research and Treatment Clinical Research Site, YRGCARE Medical Centre, Voluntary Health Services, Chennai, India;; ¶ Department of Internal Medicine, Charlotte Maxeke Johannesburg Academic Hospital and Faculty of Health Sciences, University of the Witwatersrand, Johannesburg, South Africa;; # HIV, Bill and Melinda Gates Foundation, Seattle, WA;; ** Clinton Health Access Initiative, Boston, MA; and; †† Julius Global Health, Julius Center for Health Sciences and Primary Care, University Medical Center Utrecht, Utrecht University, Utrecht, the Netherlands.

**Keywords:** tenofovir, stavudine DEXA, HIV, India, renal, South Africa, toxicity, trial, Uganda, dose reduction, resource allocation, public health

## Abstract

**Background:**

Reducing doses of antiretroviral drugs, including stavudine (d4T), may lower toxicity, while preserving efficacy. There are substantial concerns about renal and bone toxicities of tenofovir disoproxil fumarate (TDF).

**Setting:**

HIV-1–infected treatment-naive adults in India, South Africa, and Uganda.

**Methods:**

A phase-4, 96-week, randomized, double-blind, noninferiority trial compared d4T 20 mg twice daily and TDF, taken in combination with lamivudine (3TC) and efavirenz (EFV). The primary endpoint was the proportion of participants with HIV-1 RNA <50 copies per milliliter at 48 weeks. Adverse events assessments included measures of bone density and body fat. The trial is registered on Clinicaltrials.gov (NCT02670772).

**Results:**

Between 2012 and 2014, 536 participants were recruited per arm. At week 96, trial completion rates were 75.7% with d4T/3TC/EFV (n = 406) and 82.1% with TDF/3TC/EFV (n = 440, *P* = 0.011). Noncompletion was largely due to virological failure [6.2% (33) with d4T/3TC/EFV versus 5.4% (29) with TDF/3TC/EFV; *P* = 0.60]. For the primary endpoint, d4T/3TC/EFV was noninferior to TDF/3TC/EFV (79.3%, 425/536 versus 80.8% 433/536; difference = −1.49%, 95% CI: −6.3 to 3.3; *P* < 0.001). Drug-related adverse event discontinuations were higher with d4T (6.7%, 36), than TDF (1.1%, 6; *P <* 0.001). Lipodystrophy was more common with d4T (5.6%, 30) than TDF (0.2%, 1; *P <* 0.001). Creatinine clearance increased in both arms, by 18.1 mL/min in the d4T arm and 14.2 mL/min with TDF (*P* = 0.03). Hip bone density measures, however, showed greater loss with TDF.

**Conclusions:**

Low-dose d4T combined with 3TC/EFV demonstrated noninferior virological efficacy compared with TDF/3TC/EFV, but mitochondrial toxicity remained high. Little renal toxicity occurred in either arm. Implications of bone mineral density changes with TDF warrant investigation.

## Abstract 2

Supplemental Digital Content is Available in the Text.

## INTRODUCTION

For over a decade, World Health Organization (WHO) guidelines have advocated the use of a combination of 2 nucleoside analogue reverse transcriptase inhibitors (NRTIs) and a non-nucleoside reverse transcriptase inhibitors in first-line antiretroviral treatment (ART) of HIV infection.^1,2^ Over this period, toxicity concerns arose with several recommended agents, especially with the NRTI thymidine analogue stavudine (d4T).^3,4^ In 2010, WHO recommended phasing out of stavudine, due to the high incidence of serious and often irreversible mitochondrial toxicity, including lipodystrophy, peripheral neuropathy, and lactic acidosis.^5^ Because of d4T's low cost and widely available coformulation with other ART drugs, the drug had been the most prescribed antiretroviral, after lamivudine (3TC), in the world but was rapidly replaced with tenofovir disoproxil fumarate (TDF).^2,6^

Dose finding for many licensed antiretrovirals was achieved through identification of the maximum-tolerated dose that results in HIV virological suppression.^7^ Pharmaceutical companies focus on optimizing the chances of success of registration studies, titrating doses against biological markers such as CD4 cell count and viral load, generally over 48–96 weeks. The initial dose of d4T used was 40 mg twice daily (BD) for most adults (30 mg BD for patients weighing under 60 kg).^8^ Studies involving substantial numbers of patients suggested that lower doses were less toxic, while maintaining viral suppression,^9–12^ and, in 2007, WHO recommended a weight-independent reduction to 30 mg BD for all adults and adolescents.^13^ Further data, although limited, suggested that d4T doses even lower than 30 mg might maintain virological suppression, while reducing drug toxicity.^14–19^

The d4T 40-mg dose has been compared head-to-head with TDF in a trial including mostly white males in the United States,^20^ which showed no difference in virological suppression or incidence of resistance after 3 years. Lipid profiles, however, were better with TDF, and lipodystrophy, the highly stigmatizing loss of fat in the limbs and trunk, was strongly associated with d4T, affecting 19% of patients compared with 3% in the TDF arm.

Conversely, TDF has well-described renal and bone effects.^1,21^ Renal toxicity, although unusual, is potentially catastrophic in settings where renal replacement therapy, such as dialysis, is limited. The impact of TDF on bone mineral density is also well-described in dual-energy x-ray absorptiometry (DEXA) studies.^22^

At the time of initiation of the study, TDF was the most expensive component of WHO-recommended first-line therapy, costing almost four-fold more than d4T.^23^ Potentially, with a dose reduction, the overall burden toxicities with d4T could approximate those of TDF. This would raise important questions around how to balance the relative costs of the drugs and of treating the toxicities, as well as potential impacts on patient wellbeing of the 2 drugs. Although studies in India and South Africa indicate that TDF has cost effectiveness benefits when measured against conventionally dosed d4T,^24–26^ these findings may not hold true against lower doses of d4T. We thus evaluated low-dose d4T (20 mg BD) against the current gold-standard TDF, for virological efficacy and toxicity, in 3 lower- and middle-income countries (LMICs), with an emphasis on monitoring renal and bone toxicity.

## METHODS

### Study Design

This study, WRHI001, was a randomized, phase 4, multicenter, parallel-group, noninferiority trial to assess the efficacy and safety of treatment with either d4T (20 mg BD) or TDF (300 mg daily) administered in combination with 3TC (150 mg BD) and efavirenz (EFV) (600 mg daily) over 96 weeks in patients with HIV-1 infection. Participants were recruited from a clinical trial site in Johannesburg, South Africa (n = 600), in Kampala, Uganda (386), and in Chennai, India (86). The study was approved by relevant country research regulatory and ethics review bodies, and registered on Clinicaltrials.gov (identifier NCT02670772).

### Participants

Patients were 18 years and older (and younger than 65 years in the India site) and antiretroviral-naive with plasma HIV RNA levels >1000 copies per milliliter. Exclusion criteria included any exposure to ART (including mother-to-child prophylaxis), pregnancy (or desire for pregnancy in next 2 years), symptomatic peripheral neuropathy, CD4 >350 cells/µL (the threshold for ART initiation in each country at that time), hepatitis B antigen positivity, or an estimated glomerular filtration rate <60 mL/min based on the Cockcroft–Gault equation. Tuberculosis was not an exclusion criterion. As part of the informed consent procedures, participants were given detailed information on the relevant toxicities of both drugs and on the toxicity equipoise of the drugs. Participants only provided written informed consent after having taken the form home for perusal, and having had group, as well as one-on-one discussions on the study procedures and risks of participation.

### Randomization and Masking

Participants were randomly assigned in a 1:1 ratio to either d4T/3TC/EFV, “the d4T arm,” or TDF/3TC/EFV, “the TDF arm.” The randomization schedule was generated using SAS software (SAS Institute Inc, Cary, NC) and stratified by country to yield a balanced distribution of patients in the 2 arms within each site. Randomization was managed through a telephone-activated interactive voice response system. Study medications were not coformulated and were administered in a double-blind, double-dummy design. Other antiretrovirals were administered in an open-label fashion.

### Procedures

The study had separate screening and baseline visits, and 9 scheduled study visits. Women who became pregnant could elect to continue participation. Participants who developed persistent virological failure (>1000 copies per milliliter on at least 2 measures) were withdrawn from the study.

Safety evaluations included the monitoring of adverse events (AEs), physical examinations, monitoring for bone density and fat changes using DEXA, and extensive laboratory assessments. Lipodystrophy was diagnosed clinically, either after a patient complaint or on clinical examination at each visit. DEXA scans, performed at baseline, and weeks 24, 48, and 96, were sent to a central imaging organization for review, and calculation of hip and lumbar spine bone mineral density, and fat measurements (limb and truncal). In women who became pregnant, the scan was deferred until after pregnancy. Investigators did not have access to DEXA results to prevent unnecessary drug discontinuation. AEs were graded according to Division of AIDS grading.^27^ Patients who developed grade 1 or 2 AEs could continue at investigator discretion. Patients with grade 3 or 4 laboratory abnormalities were to have the results confirmed, and interruption or discontinuation of study drug considered. Substitutions of 3TC or EFV were permitted at investigator discretion, in the event of toxicity.

### Outcomes

The primary endpoint was viral suppression (HIV-1 RNA levels <50 copies per milliliter) at 48 weeks, using the “snapshot approach” in an intention-to-treat analysis.^28^ Secondary endpoints compared virological suppression at other time points and viral load cut-offs, CD4 cell count recovery, treatment emergent AEs, and laboratory toxicities at 96 weeks.

### Statistical Analysis

Monitoring and support for the study were undertaken by Pharmaceutical Product Development (PPD, Wilmington, DE). Oversight of study conduct was through a dedicated Data Safety Monitoring Board that had access to unblinded data. Stopping boundaries (critical values of *P*-value) were used by the Board for making recommendations. The study was powered based on a noninferiority margin of 10%, according to the then standards of the Department of Health and Human Services.^29^

Statistical analysis was undertaken using SAS software (SAS Institute, Inc, Cary, NC) and EAST software (Cytel, Inc, Cambridge, MA). Efficacy analysis included all randomized patients, a per protocol analysis of those who received ≥80% of their doses and had an evaluable week 48 viral load, and a safety analysis including all patients who had received at least one dose of study drug. Differences in proportions of patients who achieved the primary endpoint between the groups were analyzed using the binomial noninferiority test. To adjust for the planned interim analyses, a group sequential design after the Lan-DeMets β spending approach with the O'Brien-Fleming parameter was applied to control the type-2 error rate for the primary endpoint. The proportions of patients with the primary endpoint in each treatment group, the estimates for the difference in proportion between the groups, and the corresponding noninferiority test *P*-values were presented. Between-treatment comparisons for incidence of treatment emergent AEs were performed using χ^2^ and Wilcoxon rank-sum tests, as appropriate.

## RESULTS

**FIGURE 1.**
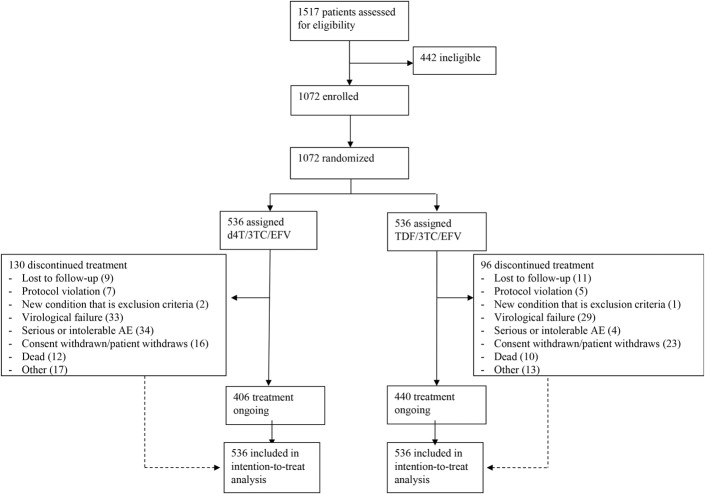
Trial profile.

Of 1517 patients screened, 1072 were randomized between July 23, 2012, and January 24, 2014 (Fig. 1). Of 536 patients in the d4T arm, 533 received at least one dose of study medication, as did 534 of 536 in the TDF arm. Week 96 completion rates were 75.7% in the d4T arm (406) and 82.1% in the TDF arm (440, *P* = 0.011). The most frequent reasons for discontinuation of the study in the d4T arm were serious or intolerable AEs [36 (6.7%)] and virological failure [33 (6.2%)]. In the TDF arm, a similar number discontinued for virological failure [29 (5.4%), *P* = 0.60], but fewer for AEs [6 (1.1%), *P* < 0.001].

Baseline characteristics are summarized in Table 1 and were similar between the 2 groups. The overall mean age of participants was 35.2 years (SD ± 8.3) and 57.7% were female. Most participants were black (91.8%, 984), with the remainder Indian. The median CD4 count at baseline was 206 cells/µL (interquartile range, 124–272). Almost 1 in 7 patients had a history of previous or current TB at study entry, nearly all pulmonary TB. Over the study, cotrimoxazole was taken by 72% of patients, isoniazid prophylaxis by 44%, and dapsone by 3%.

**TABLE 1.**
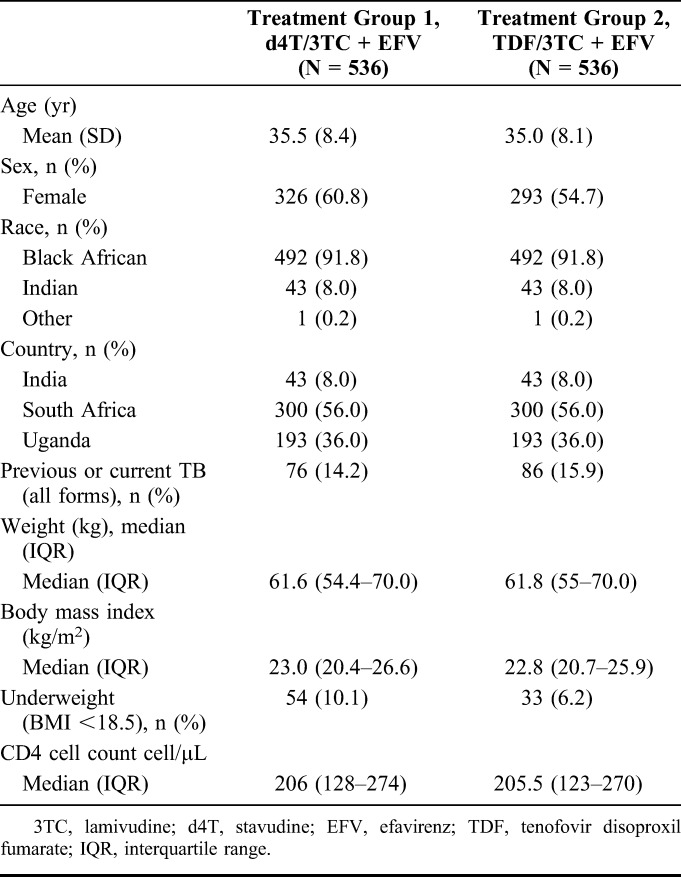
Baseline Characteristics of Participants

**FIGURE 2.**
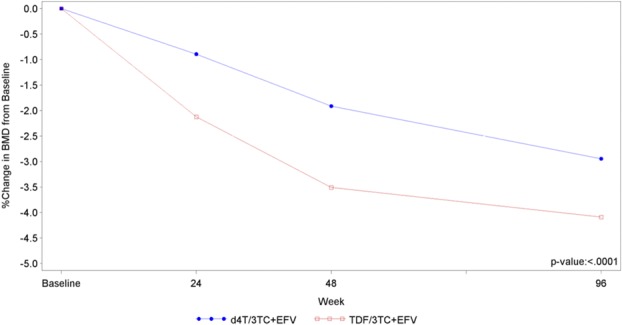
DEXA scan—hips bone mineral density percentage change from baseline.

For the primary endpoint, in the all-randomized set, the proportion of participants who had an HIV-1 RNA level <50 copies per milliliter at week 48 was similar in both arms: 79.3% in the d4T arm (425/536) and 80.8% (433/536) with TDF (Table 2). The d4T arm was noninferior to the TDF arm (treatment difference: −1.49%, 95% CI: −6.3 to 3.3; *P <* 0.001). The results of the per protocol set were similar to the all-randomized set. Similarly, the virological suppression secondary endpoints were comparable between arms, as was viral decay across all time points (see Figure 1, Supplemental Digital Content, http://links.lww.com/QAI/B241). Virological failure rates were low in both treatment groups, 43 (8.0%) patients in the d4T arm and 39 (7.3%) in the TDF arm (*P* = 0.65). There were very similar increases in CD4 cell count from baseline in both groups, over all periods, and in both the all-randomized and per-protocol set (see Figure 2, Supplemental Digital Content, http://links.lww.com/QAI/B241).

**TABLE 2.**
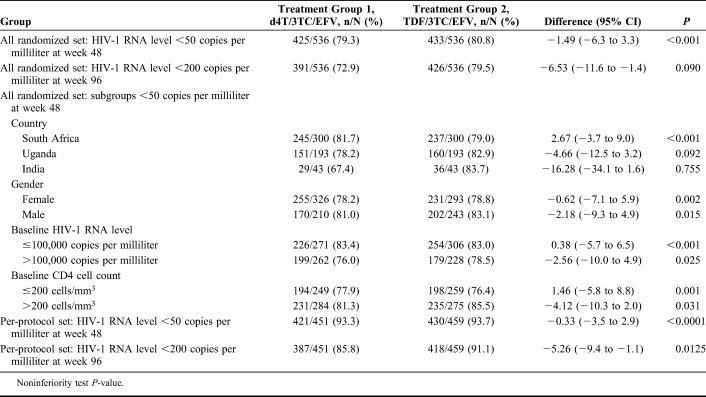
Results of all Randomized and per Protocol Analyses, by Study Arm and Population Subgroups

Results of subgroup analyses (Table 2), comparing the primary efficacy endpoint, showed that the proportion of patients with <50 copies per milliliter at week 48 was similar between the study arms when stratified by country, sex, baseline HIV-1 RNA level (> or ≤100,000 copies per milliliter), and baseline CD4 cell count (> or ≤200 cells/µL).

In aggregate, at week 48, 83.2% (480/577) of patients who had a pre-ART VL <100,000 copies per milliliter were suppressed, compared with 67.6% (378/559) if VL was >100,000 copies per milliliter (*P <* 0.001). Virological suppression was also less likely if the pre-ART CD4 cell count was <200 cells/µL. Country, TB therapy (both more and less than 3 months), a switch from EFV for toxicity, and pregnancy had no detectable impact on the rate of decrease of viral load or of reaching undetectable levels. No interaction was noted between treatment group and these associations.

Overall, 90.4% of d4T arm participants had at least one AE (482), compared with 89.0% in the TDF arm (475; *P* = 0.43). However, more d4T recipients had one or more treatment-related AE [166 (31.1) versus 129 (24.2); *P* = 0.011, Table 3]. Occurrence of at least one serious AE in the d4T arm [49 (9.2%)] was similar to the TDF arm [47 (8.8%); *P* = 0.83].

**TABLE 3.**
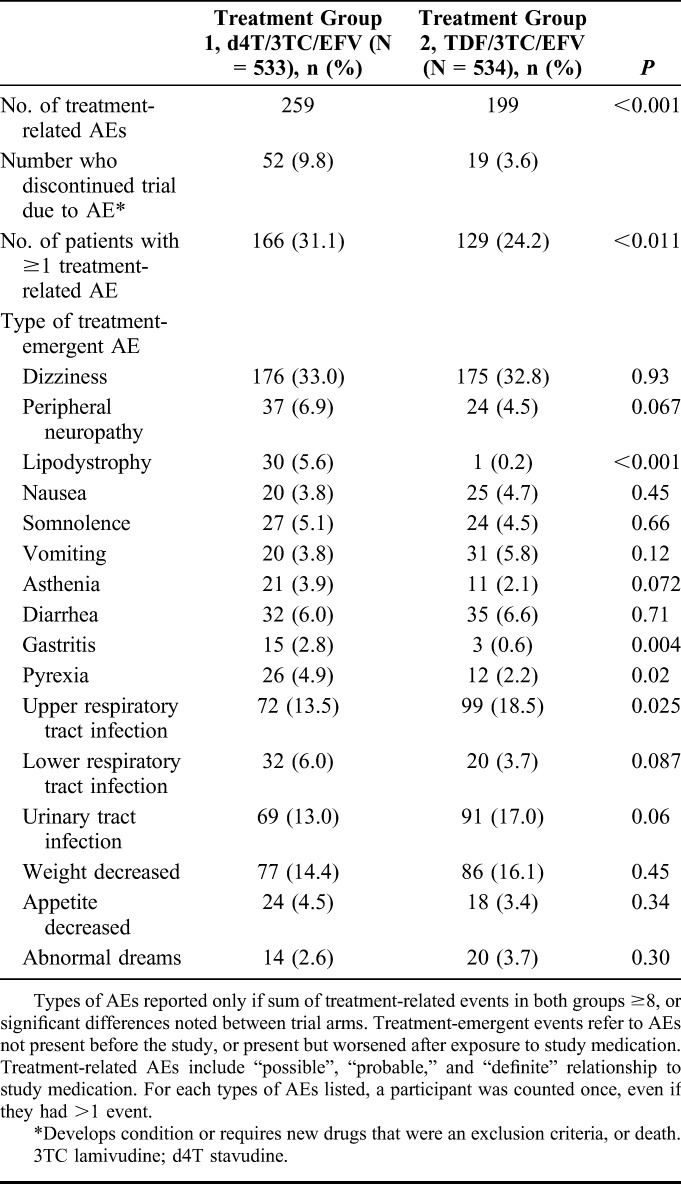
Treatment-Emergent and Treatment-Related Adverse Events

Thirteen patients died in each arm. Deaths were distributed across a broad number of causes that included infections, neoplasms, and trauma, with insufficient numbers in any category to make any meaningful inferences. No death could be ascribed to the study drugs, although cause of death was often difficult to determine because of nonspecific symptoms and the absence of an autopsy. TB was the commonest single cause of death (6 in d4T arm and 2 in TDF arm). Overall, 40 cases of TB (16 in d4T arm and 24 in TDF arm) were diagnosed during the study, including 8 cases of TB-immune reconstitution syndrome (5 in d4T and 3 in TDF arm). A total of 49 patients in the d4T arm and 36 in the TDF arm had a positive pregnancy test during the study, all of whom elected to continue in the study.

A single patient in the TDF arm developed acute renal failure, an Indian male diabetic with extrapulmonary TB, who was hospitalized and subsequently discharged with improving renal function, but then relocated and refused further contact. Four patients on the d4T arm developed lactic acidosis, all of whom recovered after discontinuation of d4T.

The incidence of the following events was higher in the d4T than the TDF arm: lipodystrophy [30 (5.6%) versus 1 (0.2%); *P* < 0.001], peripheral neuropathy [37 (6.9%) versus 24 (4.5%); *P* = 0.067], gastritis [15 (2.8%) versus 3 (0.6%); *P* = 0.004], lower respiratory tract infection [32 (6.0%) versus 20 (3.7%); *P* = 0.087], and pyrexia [26 (4.9%) versus 12 (2.2%); *P* = 0.02]. The incidence of the following events was higher in the TDF than the d4T arm: upper respiratory tract infection [99 (18·5%) versus 72 (13.5%); *P* = 0.025] and urinary tract infection [91 (17.0%) versus 68 (12.8%); *P* = 0.049]. Two patients in the d4T arm had a bone fracture and 3 in the TDF arm, whereas 5 in the d4T arm had gynecomastia as did 6 with TDF. Nine patients in the d4T arm and 20 in the TDF arm had their EFV switched due to toxicity, predominantly rash and gynecomastia (*P* = 0.039).

At 96 weeks, compared with patients in the TDF arm, those receiving d4T were more likely to have elevated levels of triglycerides (*P* = 0.017), total cholesterol (*P* = 0.006), and low density lipoprotein (LDL) (*P* = 0.039). Fasting high density lipoprotein (HDL) levels rose overall, and similarly in both arms. Lactate increased in the d4T arm over 96 weeks but did not change in the TDF arm. Seven patients in the d4T arm had their medication stopped because of raised lactate, despite being asymptomatic. There were differences between the 2 arms in several other laboratory parameters, including hematology, alkaline phosphatase, gamma globulins, and lactate dehydrogenase (see Table 1, Supplemental Digital Content, http://links.lww.com/QAI/B241). Creatinine clearance went up in both arms, overall change by end of study of 18.1 mL/min in d4T arm versus 14.2 in TDF arm (*P* = 0.03). White blood cells were detected in urine in 128 of the patients in the TDF arm and 96 of the patients in the d4T arm. One case of asymptomatic pancreatitis (grade 4 lipase and amylase) occurred in the d4T arm in a patient who had a previous history of pancreatitis and gastrointestinal TB. The patient, withdrawn from the study, recovered after study drug discontinuation. Another patient developed significant hypophosphatemia after 12 weeks, in the d4T arm, which resolved spontaneously.

**FIGURE 3.**
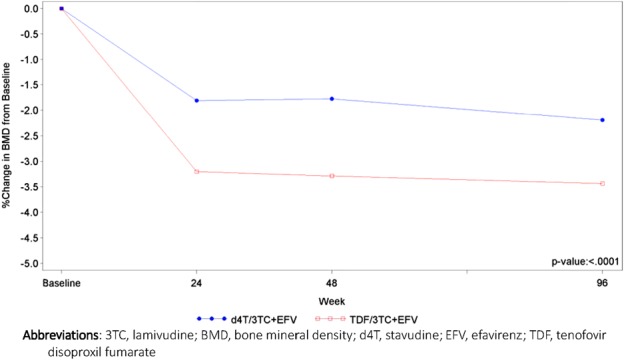
Lumbar spine bone mineral density and percentage change from baseline.

Although both arms showed a decrease in bone density from baseline, the TDF arm decrease was greater than the d4T arm for hip and lumbar spine measures (Table 4, Figs. 2 and 3). Among participants who had a hip t score of above −1 at baseline, more developed osteopenia (t <−1) during the study in the TDF arm than the d4T arm [58/391 (14.8%) versus 44/421 (10.5%); *P* = 0.06]. The median change in lumbar spine t scores from baseline was larger at weeks 24, 48, and 96. No differences were, however, observed between the study arms for lumbar spine median *t* scores for each time point, nor in the overall numbers of patients who developed osteopenia during the trial (66 in d4t arm and 71 with TDF). TDF arm patients experienced a sustained gain in DEXA-measured fat (1.18-kg gain in limb fat and 1.21 in trunk fat at week 96), whereas the corresponding figures for the d4T arm were −0.14 kg and 1.47 kg.

**TABLE 4.**
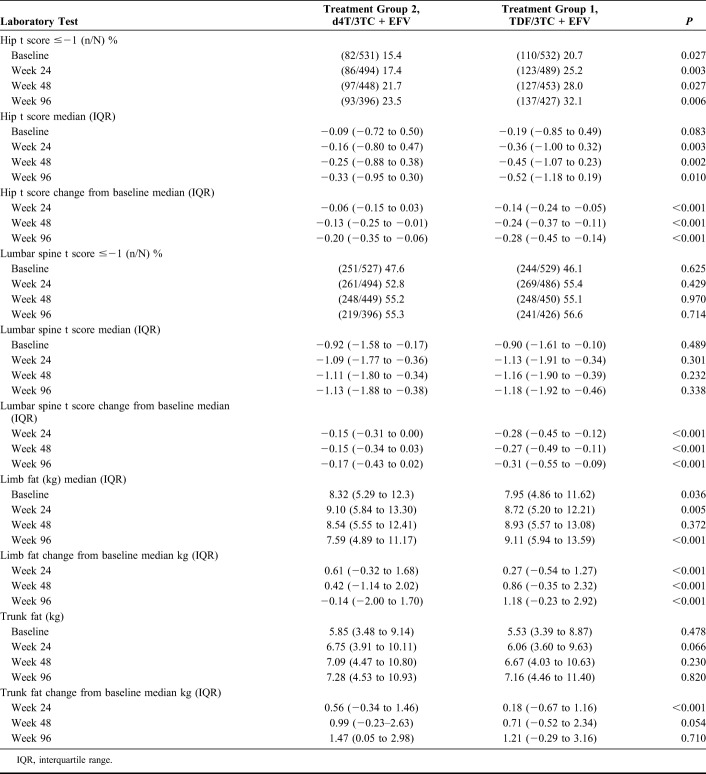
DEXA Scan Results

## DISCUSSION

The trial, the first large study to compare 20 mg BD of d4T to another antiretroviral, demonstrated low virological failure overall and noninferiority in terms of virological suppression. This suggests that d4T at 20 mg BD may have been as effective as 40 mg BD, the dose used for d4T for many years. There was, however, still substantial toxicity despite the lower d4T dose, and many more patients discontinued the study because of AEs in that arm. Generally, many of these toxicities seem to have occurred at a similar frequency to previous trials involving higher doses of the drug. However, the dose reduction may account for the lower rates of lipodystrophy with d4T in this trial than in previous studies using higher doses.^2,20^ DEXA data confirmed the fat loss, with the d4T arm reversing fat gains made in the first 48 weeks, over the subsequent life of the study, a pattern similar to earlier studies^20^·

It has been speculated that components of the lipodystrophy syndrome are due to immune reconstitution on ART.^30,31^ Our data suggest that the lipodystrophy component is due almost entirely to d4T, and not immune reconstitution, because almost all lipodystrophy events were in the d4T arm. This was significant because the study was fully blinded, and the diagnosis required either a patient-specific complaint, or clinician diagnosis. Other potential mitochondrial toxicities were noted. There was a small difference in the incidence of peripheral neuropathy, a recognized complication of d4T, and again, the differences were similar to those seen in registration studies and local evaluations.^2^ Four patients developed lactic acidosis on the d4T arm, and one pancreatitis, also recognized complications.^32^

The intensive toxicity monitoring allowed us to make a detailed assessment of the bone and renal toxicities of TDF. This is currently very topical because TDF may be phased out in favor of recently licensed tenofovir alafenamide (TAF), which has shown renal and bone benefits in recent registration trials, although their clinical significance remains uncertain.^33–36^

In our study, TDF was very well-tolerated clinically, again in keeping with other studies, with only a single case of renal failure, in a diabetic with TB.^20,37,38^ There was a small increase in GFR in both arms, noting that all started with a GFR >60 mL/min. It is difficult to explain this finding, other than to speculate that renal compromise was present at baseline because of advanced disease, which recovered somewhat as their health improved. In most other studies, TDF has been shown to decrease GFR in people with normal GFRs,^37^ but clinical trial aggregated data showed no impact when combined with EFV.^22^ Of note, there were more urinary tract infections in the TDF arm and more reports of positive urine white cell counts. It is interesting to speculate that this may be abnormal urine dipstix findings, because of tubular abnormalities, that clinicians' misassigned as infections, or that they had greater urine glucose levels and hence greater risk for infection. However, we did not see any laboratory marker changes suggesting tubular dysfunction, and the only case of severe hypophosphatemia was in a d4T patient. It is difficult to make sense of some of the other clinical and laboratory toxicity data differences (for instance, more gastritis in the d4T arm, and more lower respiratory tract infections in the d4T arm, but more upper respiratory tract infections in the TDF arm). These differences were generally not large and may have been due to type 2 errors given the multiple adverse clinical and laboratory outcomes assessed.

The study had some of the most intense monitoring of bone data ever undertaken in LMIC populations and confirmed other studies showing a significant decrease in bone density with TDF, although some loss was also seen with d4T.^20,22^ The impact from TDF and d4T was higher than seen in the initial TDF studies that used DEXA measurements and assessed TDF-induced bone loss, despite less time-to-follow-up.^20,22^ Other studies have similarly shown higher bone losses than the initial studies, also over shorter periods.^35,39–41^ Reviews suggest no impact on fracture risk with a TDF/FTC/EFV combination.^22,42^ Fractures, both upper and lower limb, were documented during the study, across both treatment arms, but numbers were too small to make any meaningful inference, and longer term follow-up is needed.

The study also allowed us to evaluate the effect of ART metabolically in a predominantly African cohort. Gynecomastia occurred in 11 men. Although this effect is uncommon, it is still responsible for 11% of all AE reports to the South African National HIV and Tuberculosis Health Care Worker Hotline.^43^ EFV has been linked to elevations in glucose^44^ and lipid changes in some studies.^45–49^ In our study, there was a general rise in lipids, more notably in the d4T arm, but in the absence of a control group, it is not possible to assess the impact of EFV on this rise. No impact, however, was seen on glucose metabolism in the study. The regimens were also considerably less effective in patients with higher viral loads (and similar across both arms), an effect also seen with the non-NRTI rilpivirine, as well as triple NRTI regimens.^50^ Although this was not an aim of the study, the difference is another reason why a move to newer, potent integrase inhibitors may be important. Finally, there were relatively few incident cases of TB (40 cases) during the study, despite all sites being in high TB prevalence areas. This is probably due to the fact that staff doing TB screening at baseline were experienced in HIV care, and almost half of the patients took isoniazid prophylaxis.

Several strengths and limitations warrant mention. The trial broadly aimed to address the public health question about whether the levels of toxicity and associated costs with d4T would be comparable with the toxicities and costs of TDF. Such findings may have warranted the continued use of d4T. This was an important question because TDF was 4-fold more costly than d4T at the time, and global HIV resource allocations were increasingly constrained. Subsequent reductions in the cost of TDF and the phasing out of d4T reduce the relevance of the study. The study, however, is able to address considerable gaps in knowledge about the medium-term safety of TDF. Most especially, we were able to examine use of TDF in Black and Asian patients, who have epidemiological and genetic differences regarding renal failure profiles and bone metabolism, when compared with whites, who made up most patients in registration studies.^51^ Importantly, the study provides compelling arguments for conducting dose-reduction studies for other ARVs and for pharmaceutics more generally. Although dose reductions offer a range of benefits, caution and close monitoring during trials are required, given the potential for virological failure and resistance if the reduced doses are subtherapeutic. Overall, however, the success of dose reduction studies to date calls into question the approach used by industry to determine the optimum dose of drugs in registration studies. Of note, before the study, there had been opposition to the trial's conduct, based on concerns that d4T would be associated with very high levels of toxicity, even with the dose reduction.^52^ However, the data were not available and needed to be generated so as to inform ART selection, and is part of a legacy of dose reduction studies that includes successful attempts to dose reduce didanosine, zidovudine and EFV, and quite likely other ARVs in future.^7,53^

## CONCLUSIONS

Low-dose d4T is suppressive, but still associated with substantial and serious medium-term mitochondrial toxicity. There may be a role for temporary low-dose d4T in clinically unstable patients, until they can be moved to safer regimens, or where patent issues compromise access to other drugs. It is also conceivable that even lower doses may be equally suppressive and have less toxicity, but the availability of cheaper TDF, and its lower-cost and safer replacement, TAF, make any studies looking at this question unnecessary.

Tenofovir was very safe in terms of renal outcomes in this study, adding to the substantial body of evidence for the remarkable utility of the drug. These data are reassuring because millions of people are receiving TDF as part of their first-line regimen in both higher-income countries and LMICs. However, the DEXA data on TDF showed substantial spine and hip impact, and the implications of these concerns warrant long-term observation. In addition, the likely introduction of dolutegravir to replace EFV in the near future merits study of the renal and bone impacts of these new combinations.

## Supplementary Material

SUPPLEMENTARY MATERIAL
