# Supplementary material for: Efficacy and Safety of Tenofovir Disoproxil Fumarate Versus Low-Dose Stavudine Over 96 Weeks: A Multicountry Randomized, Noninferiority Trial
Source: J Acquir Immune Defic Syndr. 2018 Nov 12;80(2):224–33. doi: 10.1097/QAI.0000000000001908 (PMC6358196; doi:10.1097/QAI.0000000000001908)
Supplement: SUPPLEMENTARY MATERIAL [file qai-80-224-s001.docx]

**Supplementary Figure 1: Virological decay across arms: all randomised set**

Note: Lines appear superimposed as viral decay at each time point almost identical

**Supplementary Figure 2: CD4 count recovery across arms: All randomised set**

**Abbreviations**: 3TC, lamivudine; BMD, bone mineral density; d4T, stavudine; EFV, efavirenz; TDF, tenofovir disoproxil fumarate

Supplementary Table 1 Laboratory adverse events at week 96

| **Laboratory test** | **Treatment Group 1 d4T/3TC+EFV**  (n/N) % unless indicated | **Treatment Group 2 TDF/3TC+EFV**  (n/N) % unless indicated | ***P*** |
| --- | --- | --- | --- |
| Low calcium | (23/403) 5.7 | (35/439) 8 | 0.195 |
| High calcium | (11/403) 2.7 | (8/439) 1.8 | 0.376 |
| Low phosphate | (30/403) 7.4 | (42/439) 9.6 | 0.271 |
| High phosphate | (6/403) 1.5 | (3/439) 0.7 | 0.256 |
| Low HDL | (56/403) 13.9 | (46/439) 10.5 | 0.129 |
| High HDL | (125/403) 31 | (115/439) 26.2 | 0.122 |
| Low LDL | (25/400) 6.3 | (38/435) 8.7 | 0.174 |
| High LDL | (120/400) 30 | (103/435) 23.7 | 0.039 |
| High cholesterol | (112/402) 27.9 | (87/438) 19.9 | 0.006 |
| High triglycerides | (85/403) 21.1 | (65/439) 14.8 | 0.017 |
| High lactate | (65/405) 16 | (36/437) 8.2 | <0.001 |
| Low glucose | (14/404) 3.5 | (12/438) 2.7 | 0.543 |
| High glucose | (14/404) 3.5 | (23/438) 5.3 | 0.207 |
| HOMA-IR median (IQR) | 0.11 (0.08-0.15) | 0.12 (0.09-0.18) | <0.001 |
| Low insulin | (160/248) 64.5 | (188/272) 69.1 | 0.265 |
| High insulin | (16/248) 6.5 | (19/272) 7 | 0.808 |
| High ALT | (63/403) 15.6 | (101/438) 23.1 | 0.007 |
| High AST | (59/403) 14.6 | (97/439) 22.1 | 0.005 |
| High LDH | (186/403) 46.2 | (59/438) 13.5 | <0.001 |
| Low alkaline phosphatase | (52/403) 12.9 | (34/439) 7.7 | 0.014 |
| High alkaline phosphatase | (24/403) 6 | (90/439) 20.5 | <0.001 |
| High GGT | (139/403) 34.5 | (184/439) 41.9 | 0.027 |
| Low lipase | (10/403) 2.5 | (3/439) 0.7 | 0.035 |
| High lipase | (15/403) 3.7 | (10/439) 2.3 | 0.22 |
| High gamma-globulin | (185/403) 45.9 | (231/439) 52.6 | 0.052 |
| Low creatinine | (269/403) 67.0 | (135/439) 69.3 | 0.484 |
| Creatinine median (IQR) | 43 (0.7-61) | 46 (0.8-62) | 0.049 |
| Total bilirubin median (IQR) | 3 (0.3-4.7) | 2.5 (0.3-4.5) | 0.49 |
| Total protein median (IQR) | 75 (71-79) | 75.7 (70.4-79.7) | 0.63 |
| White blood cells median (IQR) | 5.0 (4-6.2) | 4.6 (3.8-5.7) | 0.001 |
| Hemoglobin median (IQR) | 14.3 (13.3-15.3) | 14.0 (12.9-15.2) | 0.075 |
| Uric acid median (IQR) | 0.40 (0.26-3.9) | 0.38 (0.24-3.8) | 0.24 |

Cut-offs used for defining abnormal results based on laboratory values in each country. Abbreviations: 3TC lamivudine; d4T stavudine; EFV efavirenz; TDF tenofovir disoproxil fumarate. ALT Alanine transaminase; AST Aspartate transaminase; GGT Gamma-glutamyltransferase; HDL High-density lipoprotein; LDL Low-density lipoprotein;
